# Flexible Bronchoscopy in Evaluation of Persistent Wheezing in Children—Experiences from National Pediatric Center

**DOI:** 10.3390/medicina56070329

**Published:** 2020-07-02

**Authors:** Aleksandar Sovtic, Tijana Grba, Danilo Grahovac, Predrag Minic

**Affiliations:** 1 Department of Pulmonology, Mother and Child Health Institute of Serbia, 11000 Belgrade, Serbia; pbminic@gmail.com; 2 School of Medicine, University of Belgrade, 11000 Belgrade, Serbia; tijanagr@gmail.com (T.G.); graske93@gmail.com (D.G.)

**Keywords:** persistent wheezing, flexible bronchoscopy, bronchoalveolar lavage, gastroesophageal reflux, lipid laden macrophages, primary bronchomalacia, protracted bacterial bronchitis

## Abstract

*Background and objectives:* Persistent wheezing (PW) is defined as prolonged or recurrent episodes of wheezing despite regular treatment. Flexible bronchoscopy (FB) is recommended to determine the etiology of PW in children. This study aimed to determine the etiology of PW based on FB findings in a national pediatric center. *Materials and Methods:* Children presenting with PW that underwent flexible bronchoscopy from April 2016 to August 2019 at the Mother and Child Health Institute of Serbia were included in this observational study. After endoscopic evaluation, bronchoalveolar lavage fluid (BALF) samples were taken and further analyzed. Quantitative microbiology, cytological analysis and oil-red staining of specimens were performed to determine cellular constituents and presence of lipid laden macrophages (LLM). Upper gastrointestinal series were performed to exclude gastroesophageal reflux disease, swallowing dysfunction and vascular ring. *Results:* Pathological findings were revealed in 151 of 172 study participants, with bacterial lower airway infection (BLAI) (48.3%) and primary bronchomalacia (20.4%) as the most common. Younger participants were hospitalized for significantly longer periods (ρ = −0.366, *p* < 0.001). Study participants with BLAI and associated mucus plugging were notably younger (*p* < 0.001). Presence of LLM in BALF was not associated with findings of upper gastrointestinal series. All patients with confirmed BLAI were treated with oral antibiotics. Although FB is considered to be invasive, there were no complications associated with the procedure. *Conclusions:* Flexible bronchoscopy has an exceptional diagnostic value in evaluation of PW. In younger patients with BLAI, presence of mucus plugs may complicate the clinical course, so significant benefits can be achieved with therapeutic lavage during bronchoscopy.

## 1. Introduction

Wheezing is a common clinical problem in childhood. One third of all children are expected to have at least one episode of wheezing during the first three years of life, mainly as a symptom of lower respiratory tract infections [1]. Recurrent episodes of wheezing are often associated with asthma and treated with inhaled bronchodilators, inhaled corticosteroids and montelukast. However, if wheezing persists or reoccurs despite the treatment, it is defined as persistent wheezing (PW) [2]. The etiology of PW is heterogeneous and mostly age-related. Extensive workup often precedes flexible bronchoscopy (FB) in order to exclude cystic fibrosis, immunodeficiency and respiratory infections. However, FB and bronchoalveolar lavage (BAL) are recommended in order to determine the etiology of PW. Additionally, upper gastrointestinal series, a swallowing study and 24-h esophageal pH monitoring may be performed as well [2].

The primary aim of this study was to determine the causes of PW in a national pediatric center based on flexible bronchoscopy findings. In addition, contributing factors that might play a significant role in the occurrence of this condition (such as chronic lung disease of prematurity, presence of eczema and atopy in family, gastroesophageal reflux disease (GERD) and swallowing dysfunction) were also included in the analysis.

## 2. Materials and Methods

### 2.1. Study Participants

This was an observational study conducted between April 2016 and August 2019. All study participants were referred for further evaluation of PW to the Mother and Child Health Institute, a tertiary care pediatric center in Belgrade, Serbia. For the purpose of the study, PW was defined as wheezing lasting at least six weeks despite treatment. All patients with PW and humoral immunodeficiency, foreign body aspiration, cystic fibrosis and primary ciliary dyskinesia were not considered to be eligible to participate in the study. For all study participants, plain chest radiography, upper gastrointestinal series and swallowing studies were performed. Participants were discharged home with a definitive diagnosis of primary etiology of PW, in a stable clinical condition. The study was conducted in compliance with the Declaration of Helsinki. The study protocol was approved by the Local Ethics Committee (decision number 8/31).

### 2.2. Flexible Bronchoscopy and Bronchoalveolar Lavage

Bronchoscopy was performed with a standard pediatric flexible bronchoscope according to widely accepted clinical practice [3]. Each procedure was performed after an informed parental consent was obtained. Bronchoscopy was performed by one of two experienced bronchoscopists, and none of the participants had any complications after the procedure. Each bronchoscopic finding was reviewed by both bronchoscopists in order to avoid interobserver variability. Analgosedation with intravenous midazolam and epimucous lidocaine application preceded each procedure. In all patients, the nasal route was used. After an inspection of the upper airways, the procedure was continued through the vocal cords to evaluate the lower airways. Lidocain solution was instilled into the trachea to diminish cough reflex. General anesthesia was not used in order to preserve the functional mobility of the airways during breathing and coughing.

#### 2.2.1. Interpretation of Bronchoscopy Findings

Bronchoalveolar lavage was performed by the instillation of prewarmed sterile normal saline solution, by occluding the bronchus in a wedge position, followed by the aspiration of bronchoalveolar lavage fluid (BALF) from lower airways. The second collected aliquot was used for microbiological analysis and the third aliquot was considered to be representative for cellular study. The amount of lavage fluid was 10 mL per aliquot for children younger than six years of age and 20 mL per aliquot for children over six years of age. Processing of BALF specimens was performed as recommended by the European Respiratory Society Task Force on bronchoalveolar lavage in children [4]. More than 10% of neutrophils in BALF was considered to be a significant neutrophilic inflammation [5]. Subsequently, cytological analysis and oil-red staining of specimens were performed to determine cellular constituents and presence of lipid laden macrophages (LLM). Aspiration into the airway was diagnosed according to a previously proposed higher cut off value of LLM index (LLMI) [6], in the presence of clinical and radiological findings consistent with aspiration.

Quantitative BALF culture was done as well. More than 10^4^ CFU/mL of BALF was considered to indicate bacterial infection. We considered that participants with PW and positive quantitative cultures had a bacterial lower airway infection (BLAI).

Tracheomalacia was defined as a condition of excessive tracheal collapsibility with either the anterior or the posterior wall involved. Bronchomalacia was defined as excessive bronchial collapsibility [7].

#### 2.2.2. Interventions

All participants with confirmed BLAI were treated with oral antibiotics. All bacteria isolated from BAL fluid were susceptible to amoxicillin/clavulanate, which was given via oral route to each participant for two consecutive weeks.

In the case of mucus plug formation obstructing segmental or subsegmental airways, therapeutic lavage with prewarmed saline and aspiration during bronchoscopy was done. This allowed for air entry in the affected lobes and the proper inspection of the airways, distally of the obstructed site.

### 2.3. Statistical Analysis

Statistical analyses were performed using SPSS for Windows, version 21. To assess the differences between participants with normal and pathological bronchoscopy findings, as well as the differences between participants with individual endoscopy findings, a chi–square test was used. In cases where numerical conditions were not fulfilled, Fisher’s exact test was appropriate. Mann–Whitney U test was used for the analysis of ordinal data. The correlation between data was analyzed using Spearman’s correlation coefficient. The results were displayed numerically, as means ± standard deviation (SD). In all analyses, significance levels were set at 0.05.

## 3. Results

A total of 172 children with PW were enrolled, 98 (57%) males and 74 (43%) females (Supplementry material). The mean age of participants was 30.9 ± 44.6 months (median 9.6 months, range 1.5–205.2 months). The median of hospitalization length was 6 days (range 1–15 days). The characteristics of study participants are presented in Table 1.

### 3.1. Bronchoscopy Findings

Flexible bronchoscopy findings are shown in Table 2. Abnormal bronchoscopy findings were present in 151/172 (87.8%) participants, with BLAI and primary bronchomalacia as the most frequent. In 3/35 participants, primary bronchomalacia was part of a syndrome (two participants with Down syndrome and one with mucopolysaccharidosis). It was shown that pathological findings were more frequent in participants born preterm with low birth weight (*p* = 0.031).

Of the total number of participants with pathological bronchoscopic findings, 48.3% had BLAI. These participants were of significantly older age (*p* = 0.004) and more frequently had eczema (*p* = 0.032). Eight participants had primary bronchomalacia and associated BLAI. Bronchoscopy findings showed not to be associated with the presence of atopy in family and use of infant milk formulas. Moreover, occurrence of PW was not influenced by unfavorable perinatal events, such as sepsis or the use of mechanical ventilation, except for one participant with surgically repaired tracheoesophageal fistula, in whom bronchial stenosis was found. Bronchoscopy findings in children with PW in previously conducted studies are shown in Table 3.

The results of BALF cellular analysis in 87/172 (50.6%) participants showed significant neutrophilic inflammation, with normal eosinophil count. BAL findings from previously published studies are presented in Table 4.

### 3.2. Upper Gastrointestinal Series and BALF Findings

Pathological findings on upper gastrointestinal series that indicated the presence of GERD were found in 23 (13.4%) participants. The swallowing studies showed positive findings in 18 (10.5%) participants. There were no statistically significant differences between participants with different endoscopic findings with regard to results of upper gastrointestinal series and swallowing studies. Presence of significantlly elevated LLMI was found in 36/172 (20.9%) BALF specimens. Furthermore, LLMI showed no relation to pathological findings of upper gastrointestinal series. Participants with GERD had dominant neutrophilic inflammation in BALF in 17 (74%) specimens, more frequently than others (*p* = 0.035). Regardless of presence of GERD, there was no difference between groups in frequency of positive BAL cultures (*p* = 0.21). There was no difference between participants with and without GERD regarding frequency of positive BAL cultures (*p* = 0.21).

### 3.3. Hospitalization Length

**Figure 1:**
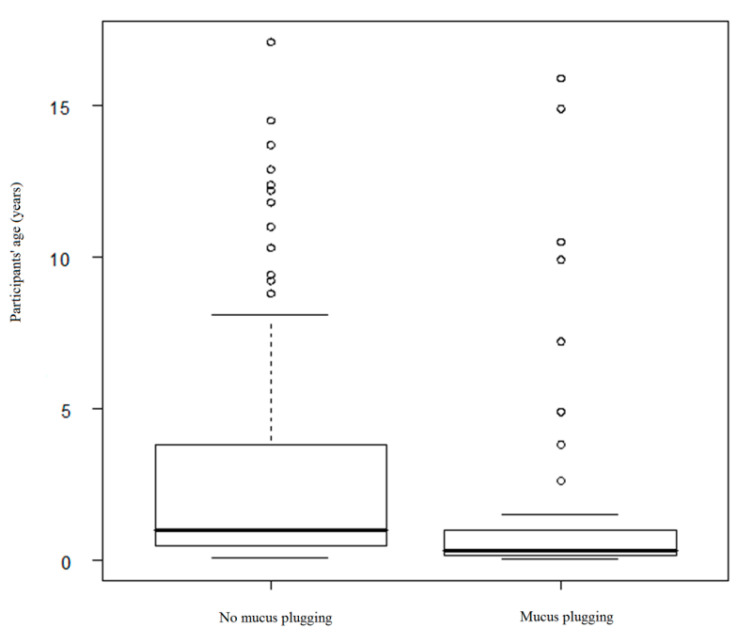
Relation of participants’ age and mucus plugging finding.

The presence of mucus plugs obstructing segmental bronchi (*p* < 0.001) was related to longer hospitalization. Moreover, it was noticed that these participants were of significantly younger age (*p* < 0.001) (Figure 1).

**Figure 2:**
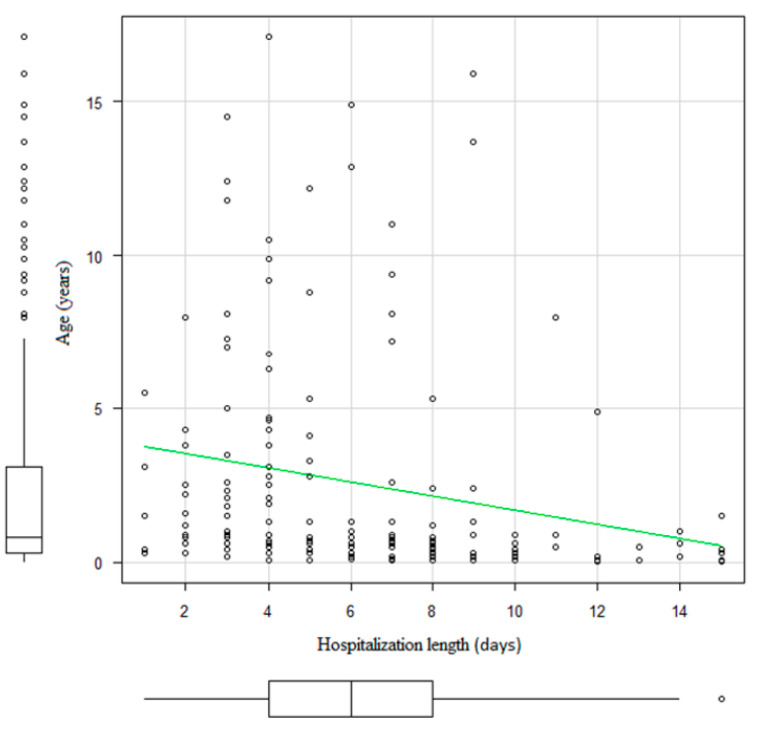
Relation of participants’ age and hospitalization length.

After correlation analysis of participants’ age and length of hospitalization was done, it was determined that younger participants were hospitalized for a longer period (ρ = −0.366, *p* < 0.001) (Figure 2).

## 4. Discussion

In the majority of patients with PW, flexible bronchoscopy leads to a definitive diagnosis, particularly if the underlying causes are congenital anomalies of the respiratory tract [7,11]. The results of our study showed that 87.8% of participants with PW have pathological bronchoscopy findings, with BLAI and primary bronchomalacia being the most frequent.

Twenty percent of children with PW in our study were diagnosed with bronchomalacia. Primary bronchomalacia is the most frequent congenital anomaly of lower airways aside from tracheomalacia [3]. It may be either isolated or occur as part of a syndrome and in most cases has a benign clinical course requiring no treatment. Bronchomalacia is sometimes complicated with chronic bacterial infection and neutrophilic BALF inflammation, as presented in some of our participants. Although most of the children with recurrent or persistent wheezing are treated with bronchodilators, those with primary bronchomalacia may have intensification rather than a decrease in wheezing intensity, as a result of accentuated dynamic collapse of affected airways. Enhancement of mucociliary clearance in order to avoid chronic respiratory infection is of paramount importance [7].

Not only is flexible bronchoscopy regarded as a diagnostic procedure, but it also has therapeutic applications [16]. During bronchoscopy, bronchial secretion and mucus plugs are aspirated from the inflamed bronchi, which leads to reventilation of atelectasis and finally reduces the length of hospitalization and medical expenses. In order to confirm this hypothesis, a controlled study would be necessary, and it was not done in this research. Prevalence of bronchitis, extramural bronchial compression and normal bronchoscopy findings are consistent with previously conducted studies [8,12,14]. None of the participants had iatrogenic granulation located in the lower respiratory tract, but its frequency was significantly higher in the aforementioned studies.

Presence of BLAI was confirmed by positive microbiological cultures in participants with endobronchial signs of inflammation (mucosal erythema, fragility and bleeding, mucus hypersecretion) and neutrophilia in BALF. BLAI complicated with mucus plugs was significantly more frequent in younger participants. It can be assumed that this is the result of smaller airway diameter, the absence of collateral ventilation of pulmonary lobules and compromised mucociliary clearance due to increased respiratory tract collapsibility during expiration [10]. Interestingly, in the clinical spectrum of chronic bacterial lower airway infections, chronic wet cough is the most prominent symptom [17,18]. However, in small children, it can often be misdiagnosed as asthma, although it can complicate its clinical course, making it difficult to treat.

Previous studies showed that microbiological cultures are positive in 40–60% of children with PW, which justifies further antibiotic treatment [5,13,15,19,20]. According to the ATS recommendation, BALF analysis should be performed in order to understand the type of inflammation in lower airways [2,4]. We demonstrated significant correlation between the presence of neutrophils in BALF, positive bacterial culture and the use of antibiotic therapy in the older age group. Overall, in our cohort, only 23% of participants had normal flora, with almost one third having two microorganisms isolated. In the authors’ opinion, it seems reasonable to treat all children with PW with antibiotics prior to an invasive procedure such as bronchoscopy, which should be done in cases of relapse or inefficient antibiotic therapy. This might be of particular importance in countries with limited availability of flexible bronchoscopy.

In 13.4% of our participants, by the means of upper gastrointestinal series GERD was found. High-resolution impedance manometry and 24-h esophageal pH monitoring are proven to have a higher sensitivity than upper gastrointestinal study in the confirmation of this condition [21,22]. Saglani et al. showed in their study that among 47 participants, two thirds had GERD [15]. Therefore, it can be assumed that the number of participants with undiagnosed GERD in our cohort is higher.

The utility of LLMI is controversial since published studies have used different diagnostic cutoff scores. Consistent with the results of Furuya et al. [6], who suggested higher values of LLMI, we decided that LLMI above 165, associated with clinical and radiography findings, was proof of chronic aspiration. Presence of high LLMI was found in 40% of participants with GERD, who also had predominant neutrophilic inflammation in BALF. This confirms that the presence of LLM in BALF does not necessarily indicate GERD, which is in line with previous studies [23,24]. The reason for this can also be swallowing dysfunction, which was not shown by our results.

Nevertheless, this study has several limitations. Firstly, the absence of a control group: Therapeutic lavage during bronchoscopy might be necessary for all patients, regardless of randomization of the procedure. Taking this into account, it was very difficult to define a control group, although international guidelines emphasize the necessity of case-control studies. In addition, a cohort study with different age groups would lead to an answer regarding whether the frequency of different findings causes PW changes over childhood age.

## 5. Conclusions

Flexible bronchoscopy has an exceptional diagnostic value in evaluation of PW etiology. Primary bronchomalacia and BLAI are the most prevalent causes of PW. According to the results of our study, PW may be an important, although atypical, symptom of BLAI. In younger patients with BLAI, the presence of mucus plugs may complicate the clinical course, so significant benefits can be achieved with therapeutic lavage during bronchoscopy. Empiric antibiotic therapy is harmless and inexpensive; therefore, sometimes it is a reasonable alternative to invasive procedures, especially if chronic wet cough is associated with PW.

## Figures and Tables

**Table 1:** Baseline characteristics of study participants.

Sex, *n* (%)	
Male	98 (57)
Female	74 (43)
Age in months, mean ± SD	30.9 ± 44.6
Hospitalization length in days, median (range)	6 (1–15)
Gestational age, *n* (%)	
<36 g.w.	22 (12.8)
>36 g.w.	140 (81.4)
Birth weight, *n* (%)	
<2500 g	51 (29.7)
>2500 g	107 (62.2)
Perinatal events, *n* (%)	
Esophageal atresia with TOF	1 (0.6)
Sepsis	6 (3.5)
Mechanical ventilation	11 (6.4)
Apgar score, median (range)	9 (2–10)
Breastfeeding, *n* (%)	78 (45.3)
Eczema, *n* (%)	22 (12.8)
Atopy in family, *n* (%)	51 (29.7)
Therapy, *n* (%)	
Systemic corticosteroids	132 (76.7)
Inhaled bronchodilators	109 (63.4)
Oxygen therapy	52 (30.2)
3% NaCl	27 (15.7)
Antibiotics	106 (61.6)
Inhaled corticosteroids	146 (84.9)

g.w.—gestational week; TOF—tracheoesophageal fistula.

**Table 2:** Flexible bronchoscopy findings.

Bronchoscopy Finding, *n* (%)	
Normal	21 (12.2)
BLAI	83 (48.3)
Bronchomalacia	35 (20.4)
Tracheomalacia	5 (2.9)
Mucus plugging	48 (27.9)
Bronchial stenosis	7 (4.1)
Extramural compression	11 (6.4)
Microbiology isolates, *n* (%)	
Normal flora	40 (23.3)
Haemophilus influenzae	38 (22.1)
Streptococcus pneumoniae	36 (20.9)
Staphylococcus aureus	17 (9.9)
Moraxella catarrhalis	25 (14.5)
Other	36 (20.9)
Upper gastrointestinal series finding, *n* (%)	
Normal	149 (86.6)
Pathological	23 (13.4)
Swallowing study finding, *n* (%)	
Normal	154 (89.5)
Pathological	18 (10.5)

BLAI—bacterial lower airway infection.

**Table 3:** Comparison of papers published on flexible bronchoscopy findings in children with persistent wheezing.

	Boesch et al. [8]	Saito et al. [9]	Wang et al. [10]	Cakir et al. [11]	De Baets et al. [12]
Number of participants	94	19	246	113	124
Sex, male %	62 %	63 %	59 %	63 %	53 %
Age, mean (range)	3.3 y (3 m–18 y)	12.5 m (5 m–26 m)	17 m (1 m–9 y)	14 m (7 m–44 m)	10 m (7 m–14 m)
Normal bronchoscopy finding, *n* (%)	12 (11.9)	0 (0)	▪	59 (52)	24 (19.4)
Bronchitis, *n* (%)	50 (49.5)	8 (42)	▪	▪	79 (63.7)
Bronchomalacia, *n* (%)	10 (9.9)	▪	▪	7 (6.2)	11 (9)
Tracheomalacia, *n* (%)	18 (17.8)	▪	▪	18 (15.9)	24 (19.4)
Mucus plugging, *n* (%)	▪	▪	52 (21.1)	▪	▪
Foreign body, *n* (%)	1 (1)	1 (5)	22 (8.9)	14 (12)	▪
Bronchial stenosis, *n* (%)	1 (1)	▪	15 (6.1)	▪	▪
Extramural compression, *n* (%)	15 (14.8)	▪	▪	2 (2)	4 (3)

▪—no data.

**Table 4:** Comparison of papers published on bronchoalveolar lavage fluid (BALF) analysis in children with persistent wheezing.

	Wang et al. [10]	Saito et al. [9]	Le Bourgeois et al. [5]	De Schutter et al. [13]	Schellhase et al. [14]	De Baets et al. [12]	Saglani et al. [15]
Number of participants	246	19	83	33	30	124	47
Sex, males %	59%	63%	70%	64%	▪	53%	53%
Age, mean (range)	17 m (1 m–9 y)	12.5 m (5–26 m)	11.3 m (4–32 m)	10 m (4–38 m)	0–18 m	10 m (7–14 m)	26 m (5–58 m)
BAL performed, *n*	98	19	83	33	27	124	44
BAL culture done, *n*	98	18	30	33	27	124	44
Positive BAL culture, *n* (%)	17 (17.3)	11 (61)	18 (60)	16 (48.5)	3 (11.1)	69 (56)	12 (27)
*H. influenzae*, *n* (%)	0 (0)	5 (27.8)	9 (30)	10 (30.3)	1 (3.7)	35 (28)	8 (18)
*S. pneumoniae*, *n* (%)	0 (0)	4 (22.2)	4 (13.3)	4 (12.1)	0 (0)	16 (13)	1 (2)
*M. catarrhalis*, *n* (%)	0 (0)	8 (44.4)	3 (10)	4 (12.1)	2 (7.4)	63 (51)	5 (11)
*S. aureus*, *n* (%)	0 (0)	▪	3 (10)	1 (3)	0 (0)	12 (10)	1 (2)
↑ LLMI, *n* (%)	▪	▪	▪	▪	5 (18.5)	▪	10 (24)
Neutrophilic inflammation, *n* (%)	▪	9(47)	▪	24/28 (85.7)	▪	▪	16/37 (43)

▪—no data; BAL—bronchoalveolar lavage; LLMI—lipid laden macrophage index.

## Supplementary Materials

The following are available online at https://www.mdpi.com/1010-660X/56/7/329/s1, Table S1. Original dataset.
